# Quality of Vitamin B12 Supplements Regarding Vitamin Assay and Content of Heavy Metals

**DOI:** 10.3390/molecules30183808

**Published:** 2025-09-19

**Authors:** Magdalena Krawczyk-Coda, Agnieszka Zgoła-Grześkowiak, Ewa Stanisz

**Affiliations:** Faculty of Chemical Technology, Poznan University of Technology, Berdychowo 4, 60-965 Poznań, Poland; magdalena.krawczyk@put.poznan.pl

**Keywords:** vitamin B12 supplementation, methylcobalamin and cyanocobalamin, metal impurities, spectrophotometry, LC-MS/MS, HR-CS ET AAS

## Abstract

Vegetarians must rely on supplements to meet the recommended daily intake of vitamin B12. Therefore, it is essential to establish a rapid, inexpensive, and reliable method of determining B12 levels in order to accurately characterize and assess the quality of supplements. This study describes a methodology for quantifying vitamin B12 in the form of methylcobalamin and cyanocobalamin following 2 min of ultrasound-assisted extraction performed at pH 4. Vitamin B12 was determined using UV-Vis spectrophotometry and liquid chromatography-tandem mass spectrometry. Thus, LC-MS/MS validated the cost-effective UV-Vis method. The content and form of vitamin B12 in the tested supplements were investigated, and serious discrepancies were found in the content or form of vitamin B12 in three out of ten supplements. To verify the quality of the analyzed supplements, the presence of metal impurities (as Cd, Hg, and Pb) was also assessed using high-resolution continuum source electrothermal atomic absorption spectrometry. No risk associated with the presence of these metals has been noted. Nevertheless, our findings underscore the need for stricter quality control in supplement manufacturing.

## 1. Introduction

Modern dietary trends, such as the vegetarian or vegan diet, are becoming increasingly popular due to ethical, health, and environmental concerns. One of the main challenges for people following this diet is ensuring an adequate supply of certain nutrients, particularly vitamin B12 (cobalamin (Cbl)). The main sources of vitamin B12 are food products of animal origin, including fish, meat, poultry, eggs, as well as dairy products [1,2]. The estimated bioavailability of vitamin B12 from food varies by type of food source and the vitamin dose [3,4]. Plant foods do not naturally contain this micronutrient. Therefore, people on a vegan diet are unable to obtain adequate amounts of vitamin B12 through diet alone. Currently, there are some fortified foods (e.g., breakfast cereals and nutritional yeasts) available on the market. These products can be relatively available sources of the vitamin [3]. However, many vegans feel the negative effects of vitamin deficiency and turn to supplements to complete this important micronutrient [5].

For people who follow a vegan diet, there are no clear recommendations from government agencies on the amount of vitamin B12 supplementation, but according to the literature, strict vegans are advised to supplement with at least 6.0 μg of vitamin B12 per day [1,6]. The European Food Safety Authority (EFSA) recommends an adequate daily intake (AI) of vitamin B12 of 4.0 μg per day for the European population in general. Estimated AIs range from 1.5 μg per day for infants (7–11 months) to 4.0 μg per day for children (15–17 years old) [7]. For pregnancy and lactation, additional vitamin intakes related to the accumulation of cobalamin in fetal tissues and its transfer into breast milk are considered. Thus, AIs of 4.5 and 5.0 μg per day, respectively, are proposed [1,7]. However, some authors point out that daily vitamin B12 losses in apparently healthy adults probably range from 1.4 to 5.1 μg. Based on the relationship between the ingested dose and the amount absorbed, vitamin B12 intakes needed to compensate for these daily losses seem to range from 3.8 to 20.7 μg in apparently healthy adults and elderly people, which is 1.4–8.6 times higher than the amount needed to prevent deficiency [4]. On the other hand, there is no recommended upper limit for vitamin B12 intake because it is a water-soluble vitamin, meaning that part of it is excreted in the urine. Therefore, to date, no adverse effects associated with excessive supplement intake have been reported in healthy individuals [8].

Vitamin B12 is a metal complex, constituted by a corrin ring and a central cobalt(III) ion bonded to six ligands, four of which are reduced pyrroles forming the corrin ring [7]. According to the ligands coordinated around the cobalt atom, four isomers of the vitamin can be listed, including hydroxycobalaminn (OHCbl), cyanocobalamin (CNCbl), methylcobalamin (MeCbl), and adenosylcobalamin (AdCbl or coenzyme B12) [1,9]. AdCbl and MeCbl are biochemically active forms of vitamin B12 in the human body. CNCbl is a stable synthetic form, which is added to food, supplements, and drugs [7]. Other forms of vitamin B12 in supplements are MeCbl, AdCbl, and OHCbl [3,10]. Regardless of the form, in cells, they have to be converted either to AdCbl or MeCbl [7,11]. Manufacturers advise using MeCbl, as it is a ready-to-use form of the vitamin, whereas CNCbl needs to be activated before being used in metabolism. Hence, it is recommended that vegan individuals should be instructed about vitamin B12 supplementation, the pharmaceutical forms available on the market and their actions, and how to choose the ideal plan to avoid vitamin B12 deficiency [8].

Additionally, there is a widespread notion that food supplements, which are bought without a prescription, are inherently safe. However, there is a serious risk of ingesting high amounts of various substances together with these products. They may be natural constituents, as is the case with plant toxins such as pyrrolizidine alkaloids [12,13]. They may result from contamination of the raw materials, as is the case with many dietary supplements containing elevated levels of toxic metals and metalloids [12,14,15]. These contaminants provide no therapeutic benefit to the consumers, and their levels in drugs and dietary supplements should be strictly controlled.

The determination of vitamin B12 in foods or dietary supplements is of great importance as it allows for the characterization of a diet. A correctly conducted analytical procedure enables the assessment of specific forms of the vitamin and its concentration in the context of food quality, correct labelling, and food regulation [15]. The approaches for the determination of vitamin B12 include microbiological assays, radioprotein-binding and spectrophotometric methods, optical biosensor-based immunoassays, and LC (liquid chromatography) techniques [1,16,17]. Among these techniques, high-performance liquid chromatography (HPLC) has become one of the most popular, due to its high sensitivity, accuracy, and precision. It can be coupled with UV, fluorescence, or mass spectrometry (MS) detection. Especially, the last-mentioned analytical tool adds a new value of sensitivity and reliability to the method, allowing for precise identification and quantification of vitamin B12 forms [17]. There is currently a trend towards reducing analysis costs, particularly in small, routine laboratories. Therefore, the low-cost UV-Vis method proposed in this paper seems to be an advantageous approach compared to those previously described in the literature.

Apart from active ingredients, dietary supplements may also contain undesirable contaminants, including metals. Among these metals, Cd, Pb, and Hg are important contaminants due to their negative impact even at very low concentrations [18,19,20]. Especially products containing animal- or mineral-based ingredients may contain metal impurities associated with their local environments [21,22]. Here, the use of AAS proposed in the present study seems to be an advantageous approach compared to more expensive methodologies like those using ICP-MS.

Additionally, it is currently difficult to find studies on the determination of metals and vitamin B12 in dietary supplements containing this active ingredient in the available literature. Only reports on the analysis of other food supplements for metal content could be found, which makes it difficult to discuss these data alongside the results obtained in the course of this study [12,14,23,24,25,26]. Therefore, it is necessary to focus on a more detailed analysis of vitamin B12 supplements in terms of the content of metal impurities.

The objective of this work is to carry out a comprehensive analysis of the vitamin B12 supplements available on the market, aimed mainly at people following a vegan and vegetarian diet. To the best of our knowledge, no methodology that uses both LC-MS/MS and UV-Vis has been presented in the literature for verifying the quality of dietary supplements containing vitamin B12. The developed LC-MS/MS method validates the cost-effective UV-Vis method. It shows its suitability for routine sample screening, and at the same time, the LC-MS/MS method may be used to confirm any discrepancies. The research focuses on two key aspects: determining the vitamin B12 content of these products and assessing the presence of potentially harmful contaminants such as Hg, Cd, and Pb in the supplements tested. The analysis of these aspects will allow for a comprehensive assessment of the quality of vitamin B12 supplements for vegans and vegetarians, which is crucial for the health and safety of consumers following this type of diet. This type of study, combining verification of potential risks resulting from insufficient active ingredient content and the presence of heavy metals, has not yet been conducted for supplements containing vitamin B12. Therefore, it allows us to verify whether supplements may contain discrepancies in declared B12 content and unsafe levels of heavy metals.

## 2. Results and Discussion

Supplements selected for the study contained vitamin B12, which was declared either as MeCbl or CNCbl. Therefore, a method was needed to separately determine these two forms of the vitamin. For this purpose, two techniques were chosen: easily available and inexpensive spectrophotometry (SPF) and more selective but very expensive high-performance liquid chromatography with mass spectrometric detection (LC-MS/MS). Thus, the LC-MS/MS method allowed us to verify whether the SPF method gives reliable results.

### 2.1. Selection of Analytical Conditions for the Determination of Vitamin B12

#### 2.1.1. Spectrophotometric Analysis

UV-Vis spectrophotometry is a technique that, in addition to determining individual compounds, enables the analysis of multicomponent mixtures in which the compounds being determined absorb radiation at different wavelengths. To select the wavelength of greatest intensity for MeCbl and CNCbl during spectrophotometric determination, the absorbance was measured in a wide wavelength range from 190 to 1100 nm. As a result, in the current work, multicomponent analysis was used to determine MeCbl, which absorbs light at 351 nm, and CNCbl, which absorbs at 361 nm. The measuring speed for this analysis was 50 nm s^−1^, and the integration time was 0.2 s.

#### 2.1.2. LC-MS/MS Analysis

**Figure 1:**
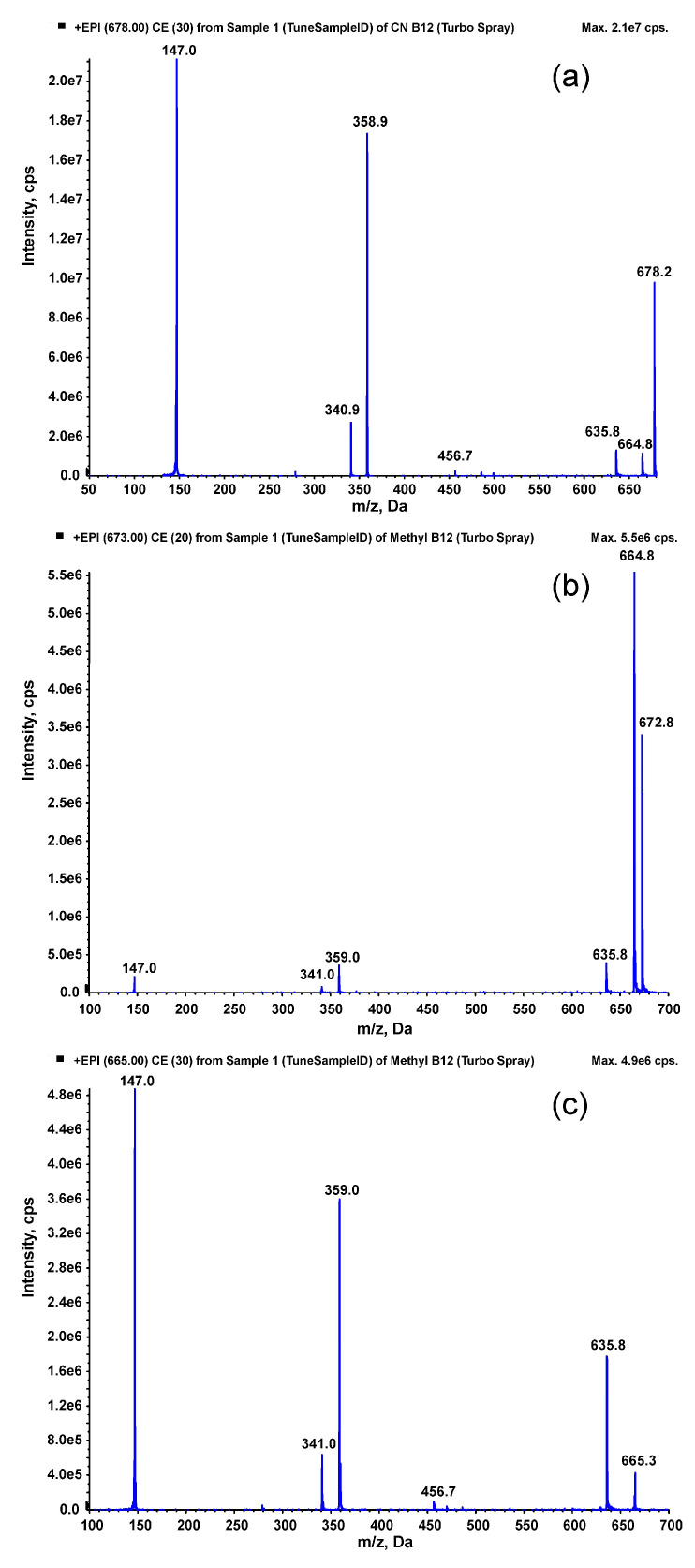
Fragmentation spectra of (**a**) cyanocobalamin (fragmentation of [M+2H]^2+^ ion at *m/z* = 678), (**b**) methylcobalamin (fragmentation of [M+2H]^2+^ ion at *m/z* = 673), and (**c**) methylcobalamin (fragmentation of the ion at *m/z* = 665).

Mass spectrometric conditions were set based on the fragmentation study of MeCbl and CNCbl. For this purpose, standards of MeCbl and CNCbl were injected directly into the mass spectrometer source using a Harvard syringe pump. Mass spectra obtained were in accordance with the literature [27], showing very intense signals from doubly protonated molecules [M+2H]^2+^ (Figure 1). For CNCbl, the ion at *m/z* = 678 was observed, which fragmented to intense ions at *m/z* = 147 and *m/z* = 359 (Figure 1a). These two transitions were selected for the determination of CNCbl (as the quantitative and confirmatory transitions). For MeCbl, the [M+2H]^2+^ ion at *m/z* = 673 fragmented to an intensive ion at *m/z* = 665 (Figure 1b), which fragmented further (Figure 1c). The transitions to *m/z* = 636 and *m/z* = 147 were selected for the determination of MeCbl (as the quantitative and confirmatory transitions). After selecting the MS/MS transitions, the chromatographic conditions were set on the HPLC system. For this purpose, an octadecylsilica column was utilized, and the mobile phase containing water/acetonitrile/ammonium formate was selected in a gradient ensuring symmetrical peak shapes and a high MS/MS signal.

### 2.2. Selection of Sample Preparation Conditions for the Determination of Vitamin B12

**Figure 2:**
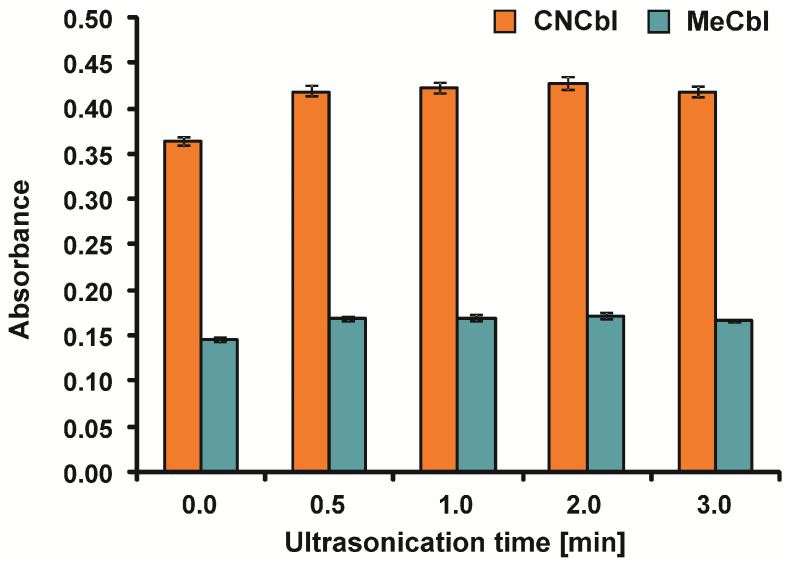
Optimization of ultrasound-assisted extraction time for MeCbl and CNCbl (n = 5).

In the course of the study, vitamin B12 was determined after ultrasound-assisted extraction. Vitamin B12 is most stable at ca. pH = 4–5 [1,3]. Therefore, the extraction was carried out at pH = 4. When synthetic vitamin B12 is added to fortified foods and dietary supplements, it is already in free form and does not require vigorous extraction. Simple aqueous extraction can be carried out. Heating may be used, and clean-up may be required to remove other analytical components [12]. Therefore, elevated temperature was not used during the tests. Instead, the ultrasound-assisted extraction of MeCbl and CNCbl was performed before their determination in vitamin B12 supplements. Therefore, the ultrasonication time was optimized to ensure maximum extraction efficiency. The study was conducted for vitamin B12 supplements C1 (CNCbl) and M2 (MeCbl). The effect of ultrasonication time was studied within the range of 0–3 min. After extraction, the sample solutions were filtered through 0.2 µm PTFE syringe filters, and both forms of vitamin B12 were determined spectrophotometrically. Maximum absorbance was achieved at a time of 2 min for MeCbl and CNCbl (Figure 2).

### 2.3. Analytical Figures of Merit for the Determination of Vitamin B12

The detection limits (LOD) calculated for the spectrophotometric determination of MeCbl and CNCbl were 250 µg L^−^^1^ and 100 µg L^−^^1^, respectively. The limits of quantification (LOQ) were 833 µg L^−^^1^ and 333 µg L^−^^1^ for MeCbl and CNCbl, respectively. The relative standard deviations (n = 5), calculated for 1 mg L^−^^1^ in a standard solution containing both forms of vitamin B12, were 1.5% and 1.2% for MeCbl and CNCbl, respectively. Calibration was performed by the standard calibration technique. The linear ranges of the calibration functions were 0.5–50 mg L^−^^1^ for both forms of vitamin B12. The working ranges of the calibration functions were 0.5–80 mg L^−^^1^ for MeCbl and CNCbl. The acceptable correlation coefficients of 0.9997 and 0.9998 were achieved for MeCbl and CNCbl, respectively.

The limits of detection for the LC-MS/MS determination of MeCbl and CNCbl were calculated as 3 times the signal-to-noise ratio, and the limits of quantification as 10 times the signal-to-noise ratio. For MeCbl, LOD was 0.2 µg L^−^^1^ and LOQ was 0.8 µg L^−^^1^, while for CNCbl, LOD was 0.01 µg L^−^^1^ and LOQ was 0.04 µg L^−^^1^. Linearity was tested at 9 concentration levels from 0.5 to 1000 µg L^−^^1^ for both analytes. Excellent correlation was found for both MeCbl (R^2^ = 0.9992) and CNCbl (R^2^ = 0.9997). The high sensitivity of the LC-MS/MS compared to the spectrophotometric determination of vitamin B12 necessitated greater dilution of the sample. However, this had a positive impact, as there was no matrix effect for the samples being tested.

Due to the lack of certified reference materials in the form of dietary supplements, the standard addition was used to ensure the accuracy of the developed procedures. For spectrophotometric determination of MeCbl and CNCbl in capsules, the average recoveries were 99.1 ± 1.2% and 98.8 ± 1.5%, respectively, and in tablets, 98.5 ± 1.5% and 98.7 ± 1.4%, respectively. The average recoveries obtained for LC-MS/MS determination of MeCbl and CNCbl in capsules were 100.6 ± 1.6% and 97.4 ± 4.5%, respectively, and in tablets were 97.7 ± 3.2% and 99.4 ± 5.1%, respectively. The recovery values are within the accuracy range proposed in the Guidelines for Dietary Supplements and Botanicals [28], which suggests a 90–108% range for supplements containing 0.1% of the active ingredient.

The matrix effect was calculated based on the ratio of the slope of the enriched sample curve to the reference curve. The calculated matrix effect proved to be negligible—below 5%.

### 2.4. Determination of Vitamin B12 in Supplements

To evaluate the usefulness of the proposed analytical procedures for the determination of MeCbl and CNCbl in vitamin B12 supplements, the contents of these analytes in ten samples were established using the experimental conditions previously optimized. Quantitation of analytes by LC-MS/MS was performed using the external standard technique, for which standards at a concentration of 0.5 µg mL^−^^1^ were used. During the spectrophotometric determination of MeCbl and CNCbl, the standard calibration technique was used. The obtained results of the analysis are given in Table 1.

One of the aims of the research presented in this paper was to assess the quality of the supplements by determining the content and typical forms of vitamin B12. Supplements containing the declared MeCbl and CNCbl contents were selected for testing. The results indicate that the forms of vitamin B12 declared by the manufacturers were consistent with those determined during testing. However, sample C1 contained significantly higher amounts of MeCbl (7.2 ± 0.5 µg and 6.1 ± 0.1 µg) and very low amounts of CNCbl (0.9 ± 0.1 µg and 0.6 ± 0.1 µg), in addition to the declared CNCbl dose of 10 µg. The declared MeCbl contents ranged from 100 µg to 500 µg per tablet/capsule, depending on the supplement. The content closest to the declared amount was found in sample M3 (97% and 94%), while the lowest amount was found in sample M1 (39% and 39%) and M4 (45% and 41%). All doses determined were lower than the declared amounts, ranging from 39% to 97%. The exception was sample M6, which contained 122% and 108% of the declared vitamin B12 content. In turn, the content of CNCbl declared in the tested samples was from 10 µg to 500 µg per tablet/capsule, depending on the supplement. The content closest to the declared amount was found in samples C2 (106% and 127%) and C3 (111% and 105%). The lowest content was determined in sample C4 (79% and 86%).

There are not many precise requirements on the content of active ingredients in supplements in relation to their labeled declaration. Very precise requirements were defined by the Canadian Food Inspection Agency, stating that each sub-sample should contain ±50% of the labeled active ingredient and ±20% in three composite sub-batches [29]. Taking into account that only one batch of each supplement was acquired in the present study, the limit ±50% has to be applied. This means that two out of ten samples tested (i.e., M1 and M4) contained too little vitamin B12. Insufficient levels of active ingredients in supplements, as well as their absence or the presence of undeclared ingredients, are a fairly common problem worldwide [30,31,32]. Unfortunately, although the introduction of new supplements must be reported to the supervisory authorities, their control is incomplete. Often, only a few percent of supplements on the market are subject to control [33,34].

Of the two forms of vitamin B12 identified during the study, MeCbl is the natural, biochemically active form found in the human body, whereas CNCbl is a stable synthetic form that is often added to supplements [7]. CNCbl is used frequently in supplements as it is considered more stable and cost-effective than other forms of vitamin B12 [35,36]. Once inside the body, CNCbl is converted into either MeCbl or AdCbl, both of which are active forms of the vitamin. Some research suggests that the bioavailability of each form may differ depending on factors such as gastrointestinal pathologies, age, and genetics [35,36]. Although some early studies found that the human body absorbed 49.2% of CNCbl (calculated for a 1 μg dose) compared to 44.4% for a 1 μg dose of MeCbl. For a higher dose of 25 µg, these values decrease to 5.6% and 6.1% for CNCbl and MeCbl, respectively [37]. Additionally, some studies suggest that, for long-term supplementation, the use of natural forms (OHCbl, MeCbl, or AdCbl) should be favored over CNCbl in order to avoid the accumulation of cyanide in the human body. This is particularly important for tobacco smokers, who are exposed to this compound through cigarette smoke [35,38,39,40]. Consequently, there is a clear trend towards replacing CNCbl supplements, which were previously almost the only option on the market, with their natural forms, especially MeCbl [35,38].

It must also be taken into account that the differences in absorption of vitamin B12 are strictly connected with the mechanisms involved. Vitamin B12 liberated from food binds with haptocorrin present in saliva and stomach fluids and moves with it through the gastrointestinal tract. Then, vitamin B12, liberated by proteolytic enzymes, is absorbed through two routes—binding to the intrinsic factor protein and diffusion through the mucosa. Unfortunately, the intrinsic factor protein becomes saturated at 2 µg per meal [35]. Moreover, there are many conditions influencing vitamin B12 absorption, including autoimmune pernicious anemia and atrophic gastritis, which both lower the production of intrinsic factor, or celiac disease, ulcerative colitis, Crohn’s disease, and tropical sprue, which reduce absorption by endocytosis [35]. Nevertheless, vitamin B12 can also be absorbed by diffusion without the need for an intrinsic factor. This mechanism, however, works only when high doses of vitamin B12 are administered [41]. Therefore, supplementing vitamin B12 often requires as much as a 1000 µg daily dose [35,41] even though the recommended daily allowance (RDA) is much lower—2.4 µg per day in the United States according to the National Institutes of Health (NIH) [3,35]. As a result, constant supplementation is crucial, and supplements of vitamin B12 with content considerably lower than declared, including samples M1 and M4 analyzed in the present study, can lead to health issues.

The European Food Safety Authority (EFSA) Panel (EU) has set an adequate daily intake (AI) of 4.0 μg per day for adults, based on data on different biomarkers of cobalamin status, and taking into account observed intakes in several EU countries, which range between 4.2 and 8.6 μg per day [7]. For healthy breastfed infants, the AI is the mean intake. For other life stages and gender groups, the AI is believed to cover the needs of all individuals in the group. However, a lack of data or uncertainty in the data prevents being able to specify with confidence the percentage of individuals covered by this intake. RDAs are set to meet the needs of almost all individuals (97–98%) in a group. A comparison of the values determined shows that they significantly exceed the values required by EU and US regulations (Table 1). These amounts exceed by a large margin the established limits of 2.4 and 4.0 μg per day, and ranged from 6.7 µg per day (sample C1) to 435.0 (sample M2) μg per day assuming that the consumer takes one tablet or capsule per day. It is significant that, in addition to the AI and RDA values, neither the EU nor the US government organizations declare the form of vitamin B12 that dietary supplements should contain.

### 2.5. Selection of Conditions for the Determination of Cd, Pb, and Hg

An important issue raised in the study is the safety of vitamin B12 supplements, specifically the presence of certain inorganic contaminants. Their determination requires the development of sample preparation methods and the selection of proper analytical conditions for the determination of elements.

#### 2.5.1. Optimization of ET AAS Detection of Cd and Pb

Before Cd and Pb determination, the samples were prepared using microwave-assisted digestion, according to the parameters given in the Materials and Methods Section. Absorbance measurements for Cd were performed at the wavelength of greatest intensity, i.e., 228.8 nm. For Pb, the wavelength of greatest intensity is 217 nm. Unfortunately, at this wavelength, phosphorus oxide can cause spectral interferences. Therefore, the absorbance for Pb was measured at 283.3 nm to obtain accurate results.

The temperature program of the electrothermal atomizer (ET) of the AA spectrometer was optimized for standard solutions containing 5 ng mL^−1^ of Cd and 20 ng mL^−1^ of Pb. Three drying steps allow for avoiding the spattering of the sample and obtaining a uniform liquid deposit on the graphite surface. The effect of pyrolysis temperature on absorbance was studied within the ranges 600–1200 °C and 800–1400 °C for Cd and Pb, respectively. The absorbance reached a maximum at 1000 °C and 1300 °C for Cd and Pb, respectively, and these values of the pyrolysis temperature were chosen for further experiments. After optimization of pyrolysis conditions, the effect of atomization temperature on analytical signals was studied within the ranges 1200–1600 °C and 1500–2300 °C for Cd and Pb, respectively. Maximum absorbances were achieved at a temperature of 1500 °C and 2200 °C for Cd and Pb, respectively. Therefore, these atomization temperatures were chosen for the determination of Cd and Pb in vitamin B12 supplements. During the experiments, chemical modifiers (magnesium for Cd and phosphate for Pb) were used to ensure stable analytical signals with minimal influence exerted by the matrix. The temperature program of the electrothermal atomizer used for Cd and Pb determination is shown in the Section 3.

#### 2.5.2. Optimization of CV AAS Detection of Hg

Mercury was determined using cold vapor atomic absorption spectrometry at a wavelength of 253.65 nm, which is commonly used for that purpose. A commercially available automatic mercury analyzer was used for the determination during the tests. The values for the most important parameters for Hg vapor generation were used as recommended by the manufacturer. Before Hg determination, the samples were prepared using microwave-assisted digestion, according to the parameters given in the Materials and Methods Section.

SnCl_2_ is the most efficient reducing agent for Hg. Therefore, a 2% (*w/v*) solution of this reagent was used. An acidic medium (2 mol L^−1^ HCl) was applied to achieve an effective and rapid reaction of vapor generation. The sensitivity was affected by the flow rates of the reducing agent, acid, and sample, since higher flow rates resulted in higher signal intensity. However, too high a flow rate can result in concentrated, unstable vapor; therefore, an ideal sample-to-reagent ratio is required for maximum vapor generation efficiency. The optimum flow rates for SnCl_2_ and HCl were 1.0 mL min^−1^. The maximum signal was obtained at a sample flow rate of approximately 1.7 mL min^−1^.

During Hg determination, the metal ions can be adsorbed onto the surfaces of the tubes and crossflow reactor, resulting in memory effects. To effectively limit this phenomenon, an appropriate flushing medium is required. During the tests, a 0.1% (*m/v*) solution of NH_4_OCl was used for this purpose. The flow rate of the carrier gas (Ar) also played an important role in the Hg cold vapor process. The argon flow rate should be high enough to strip the vapor from the crossflow reactor and carry it up to the sample cell, but not so high as to dilute the formed volatile product. The optimum argon flow rate for Hg determination was found to be 4.5 L h^−1^. Calibration was performed using the standard calibration technique with aqueous standards in an acidic medium. The standard solutions were prepared immediately prior to measurement within the concentration range of 0.1–5.0 μg L^−1^.

### 2.6. Selection of Microwave-Assisted Digestion of the Supplements

The metals present in the supplements were quantified after microwave-assisted digestion. Solid supplements (whole tablets and capsule contents) were prepared by digestion in a closed microwave system with focused energy. This type of approach to sample preparation is a widely used technique in spectroscopic analysis [42,43]. The samples were prepared using an oxidizing mixture consisting of concentrated HNO_3_ and 30% H_2_O_2_, with the addition of 40% HF. The microwave-assisted acid digestion used allows for rapid and efficient decomposition of the solid matrix with reduced acid consumption, good reproducibility, and lower contamination risk. The mass of the samples was controlled using an analytical balance and was approximately 0.3 g, which ensured stable and safe conditions.

### 2.7. Analytical Figures of Merit for the Determination of Cd, Hg, and Pb

The limits of detection for inorganic impurities were calculated as the concentration of the analyte yielding a signal equivalent to three times the standard deviation of the blank value (n = 5). The values were 0.05 µg L^−^^1^, 0.01 µg L^−^^1,^ and 1 µg L^−^^1^ for Cd, Hg, and Pb, respectively. The limits of quantification were 0.17 µg L^−^^1^, 0.03 µg L^−^^1^, and 3.3 µg L^−^^1^ for Cd, Hg, and Pb, respectively. The relative standard deviations (RSDs), calculated for five replicate measurements of 10 µg mL^−^^1^ in a standard solution containing both elements, were 7% and 5% for Cd and Pb, respectively. RSD obtained for Hg, for five replicate measurements of 0.25 µg mL^−^^1^ in standard solution, was 4%. For all three analytes, the calibration was performed by the standard calibration technique. The linear ranges of the calibration functions were 0.5–20 µg L^−^^1^ for Cd, 10–40 µg L^−^^1^ for Pb, and 0.1–5.0 µg L^−^^1^ for Hg. The working ranges were 0.5–30 µg L^−^^1^ for Cd, 5–60 µg L^−^^1^ for Pb, and 0.1–10.0 µg L^−^^1^ for Hg. The acceptable correlation coefficients (R^2^ = 0.9998 for Cd, R^2^ = 0.9995 for Pb, and R^2^ = 0.9992 for Hg) were achieved.

Due to the lack of certified reference materials in the form of dietary supplements, the standard addition was used to ensure the accuracy of the developed procedures. The standard addition method was also used to verify the accuracy of the Cd, Pb, and Hg determination procedures. The average recoveries obtained for Cd, Pb, and Hg in capsules were 97.4 ± 6.1%, 96.6 ± 5.1% and 97.2 ± 3.1%, respectively, and in tablets, 96.3 ± 5.3%, 96.6 ± 4.8% and 98.1 ± 2.5%, respectively. The results show that the proposed analytical procedures can be applied to determine inorganic impurities (Cd, Pb, and Hg) in vitamin B12 supplements. For this reason, no further studies on the matrix effect have been carried out.

### 2.8. Cd, Pb, and Hg Determination in Vitamin B12 Supplements

In the course of the study, Cd and Pb were determined in vitamin B12 supplements by HR-CS ET AAS, and Hg was quantified using CV AAS detection. The results of the analysis are presented in Table 2.

The developed methods were utilized for the determination of Cd, Pb, and Hg. These heavy metals are sometimes present in pharmaceutical products and supplements and can harm humans. Therefore, their concentrations in these products should be monitored [23]. Metals can appear in a final product through various routes. For example, they may be residual catalysts added intentionally during synthesis, impurities introduced through interactions with processing equipment or container/closure systems, or they may be present as impurities in active ingredients or excipients. Importantly, products containing ingredients of natural origin may contain heavy metals (such as Cd, Pb, and Hg) due to their accumulation from the soil, water, or air. Thus, both animal- or mineral-based dietary supplements may contain metal impurities associated with their local environments [21,22].

To address this issue, the European Medicines Agency (EMA) and the United States Pharmacopeia (USP) have developed guidelines for the risk assessment of elemental impurities [18,19,20]. These agencies have established permitted daily exposure (PDE) levels for these elements. Therefore, identifying the impurity and carrying out a risk assessment according to element classification and route of administration are easier.

The elements included in the ICH (International Conference on Harmonization) EMA guideline have been divided into three classes based on their toxicity (PDE) and likelihood of occurrence in the drug product [18]. This likelihood was determined based on various factors, including the probability of use in pharmaceutical processes and the probability of co-isolation with other elemental impurities in materials used in pharmaceutical processes. It also took into account the observed natural abundance and environmental distribution of the element. Cd, Pb, and Hg are class 1 elements, which are described as human toxicants with limited or no use in pharmaceutical manufacturing.

Additionally, the elements of toxicological concern are mentioned in Chapter 2232 of the USP [20]. According to the document, the PDE for both Cd and Pb was defined as 5 µg per day, and for Hg (total and methylmercury) as 15 µg per day and 2 µg per day, respectively.

In Europe, the EU Commission Regulation [44] sets maximum permissible levels for Cd, Pb, and Hg in various types of food. The values for Cd range from 0.005 mg kg^−1^ (in infant formulae) to 3.0 mg kg^−1^ (in food supplements consisting of at least 80% dried seaweed). Pb levels vary between 0.010 mg kg^−1^ in infant formulae and 3.0 mg kg^−1^ in food supplements, and Hg levels range from 1.0 mg kg^−1^ in muscle meat from fish to 0.10 mg kg^−1^ in food supplements.

Although the amounts of Cd and Pb determined in the supplements are greater than the European limits for infant formulae, one must take into account that these supplements are not intended for infants, and the amount of supplement taken is also restricted to one tablet (or capsule). Therefore, taking into account the amount of Cd and Pb determined and the mass of one tablet (or capsule), none of the supplements analyzed in this study exceeded the limits given in the USP guide (Table 2).

It is also difficult to draw conclusions about the presence of these elements in the supplements since concentrations of inorganic impurities differ considerably between products (and thus between studies, depending on the products selected) [12]. For example, some previous studies investigating a large number of food supplements of different origins [14,24,25,26] reported maximum concentrations of Cd, Pb, and Hg in the ranges 0.940–500 mg kg^−1^, 0.036–50 mg kg^−1^, and 0.0004–0.550 mg kg^−1^, respectively, thus varying by several orders of magnitude.

### 2.9. Hazard Quotients

Considering all available information, i.e., concentrations, number of tablets (or capsules) consumed, and the reference dose for each element, the hazard connected with the intake of supplements may be calculated and expressed as hazard quotients (HQs). The hazard quotients were calculated to assess the human health risk due to chronic exposure to Cd and Pb [14]. Values for Hg were not calculated as the content of this element was below the LOD of the analytical method used. The determined quotients show whether the average daily intake is greater than the reference dose. Thus, for HQs greater than one, there is an unacceptable risk due to chronic exposure to Cd or Pb, and adverse effects may arise, while for HQs equal to or lower than one, the risk is acceptable.

Due to both different concentrations and ingestion rates for each kind of tablet or capsule tested, the HQ values were calculated separately for each supplement. The obtained results were summarized in Table 3 and show no substantial health risk connected with the consumption of the tested products. Also, to summarize the effect of Cd and Pb, the hazard index (HI) was calculated, which is the sum of the HQ values for both metals. The HI values above 1 show unacceptable risk, and for HI ≤ 1, no significant risk is expected [45]. The results presented in Table 3 show no risk due to the consumption of the tested food supplements.

The HQ values obtained for Cd and Pb are considerably lower than one, which means that the analyzed supplements are safe for consumption. The calculated values of the hazard index show that the supplements are also safe when taking into account the combined content of determined heavy metals.

## 3. Materials and Methods

### 3.1. Reagents

Compressed argon of UHP 5.5 purity obtained from Air Products (Poznań, Poland) was employed as an inert gas without further purification. Nitrogen obtained from a generator was used as a collision gas during LC-MS/MS analysis. The analytical standards (MeCbl and CNCbl) were purchased from Merck/Sigma-Aldrich (Steinheim, Germany). Standard solutions of MeCbl and CNCbl were prepared daily by dissolution of a proper amount of the solid substances. Standard solutions of Cd, Hg, and Pb were prepared from 1000 mg L^−1^ standard solutions from NIST (Certipur, Merck, Darmstadt, Germany). Standard solutions of Hg were stabilized with K_2_Cr_2_O_7_ (5% *m/v*, GR, Merck). SnCl_2_ (2% *m/v*), used as a reducing agent, was prepared by dissolving the appropriate mass of SnCl_2_∙2H_2_O (Merck) in concentrated HCl (32% *v/v* extra pure, Merck) and by diluting to the desired volume with ultra-pure water. The HCl concentration in the reducing agent was 2 mol L^−1^. Additionally, a rinsing solution of NH_4_OCl (0.1% (*m/v*)) was used. MS-grade acetonitrile and ammonium formate were purchased from Sigma-Aldrich. Chemical modifier solutions: phosphate 100.0 ± 0.2 g L^−1^ (as NH_4_H_2_PO_4_) and magnesium 10.0 ± 0.2 g L^−1^ (as Mg(NO_3_)_2_) were used for HR-CS ET AAS detection. Additionally, 65% HNO_3_, 40% HF, and 30% H_2_O_2_ (Suprapur, Merck) were used for microwave-assisted digestion. High-purity water—deionized (DEMIWA 5 ROSA, Watek, Ledeč nad Sázavou, Czech Republic) and doubly distilled (quartz apparatus, Bi18, Heraeus, Hanau, Germany)—was used during the research. The resistivity of the water was 18 MΩ cm.

### 3.2. Vitamin B12 Supplements

In the course of the study, the analytes were determined in ten vitamin B12 supplements that were purchased from local pharmacies or online stores. According to the producers’ declaration, six supplements of vitamin B12 (M1-M6) occurred in the form of MeCbl, and four supplements (C1-C4) in the form of CNCbl. Samples M1, M5, and C1-C3 were purchased in the form of tablets. Samples M2-M3, M6, and C4 were in the form of capsules. Before the digestion and extraction procedures, the tablets/capsule contents were ground manually in a ceramic mortar and stored in polymer containers.

### 3.3. Sample Preparation

#### 3.3.1. Sample Pretreatment Before Vitamin B12 Determination

The contents of capsules and tablets of the analyzed supplements were homogenized before sample preparation. Thirty tablets or 30 capsules were ground in a porcelain mortar and mixed thoroughly. Due to the light sensitivity of vitamin B12, all activities related to the preparation of the samples and the determination step were conducted in dark conditions. In the course of preparation, step samples were weighed using an M2P microanalytical balance (Sartorius, Gottingen, Germany) with a resolution of 1 μg.

A Sonopuls HD 70 ultrasonic homogenizer (70 W, 20 kHz, Bandelin, Berlin, Germany) equipped with a 2 mm titanium microtip was used for the extraction of MeCbl and CNCbl before spectrophotometric and LC-MS/MS analysis. It was decided to use the ultrasound homogenizer as it ensured fast disintegration of tested samples regardless of their type (tablets or capsules), while the method was safer compared to the microwave-assisted extraction, which is widely used mainly in sample preparation for the determination of metals.

Prior to the determination of MeCbl and CNCbl in vitamin B12 supplements, it was necessary to extract the analytes from the ground samples using ultrasound. Whole samples of film-coated tablets were ground using a porcelain mortar, while only the contents of capsules were homogenized. Approximately 250 mg of the sample was placed in a glass vial, and 10 mL of water was added. Therefore, ultrapure water was used as a solvent during the preparation process. Ultrasound-assisted extraction was performed using an ultrasonic probe (2 mm titanium microtip, 40 W, 20 kHz) for 2 min at pH = 4. Before LC-MS/MS and spectrophotometric analysis, samples were filtered through 0.2 µm PTFE syringe filters and diluted with distilled water to bring the concentration within the linearity range of the method. Corresponding blanks were also prepared using the procedure described above. MeCbl and CNCbl were determined immediately after sample preparation.

#### 3.3.2. Sample Pretreatment Before Metal Impurities Determination

In the course of preparation, step samples were weighed using an M2P microanalytical balance (Sartorius, Gottingen, Germany) with a resolution of 1 μg.

A UniClever focused microwave sample preparation system (Plazmatronika, Wrocław, Poland) operating at 2450 MHz and 300 W maximum output was used for wet digestion of vitamin B12 supplements before Cd, Hg, and Pb determination. The computer-controlled system with continuous temperature, pressure, and microwave power monitoring was equipped with a high-pressure TFM-PTFE vessel (110 mL) and a water-cooling system. The maximum pressure and maximum temperature possible to control in the system were 100 atm and 300 °C, respectively.

As with ultrasound-assisted extraction, the film-coated tablets were ground whole. For capsules, only the contents were ground, using a porcelain mortar. Approximately 300 mg of vitamin B12 supplement was placed in the vessel of the microwave digestion system. Then, 1 mL of 30% H_2_O_2_, 5 mL of 65% HNO_3_, and 0.25 mL of 40% HF were added. The microwave power was applied for 20 min at 300 W. After digestion, the clear digested solutions were transferred to 10 mL volumetric flasks and diluted to volume with ultrapure water.

HF was used because the manufacturers of the tested dietary supplements listed talc (a silicate mineral) and silicon dioxide, among other ingredients, on the product labels. These components are most often added as fillers to achieve the desired weight, as glidants to improve the flow of ingredients into machinery, or as lubricants to prevent tablet and capsule shells from sticking to machinery. Therefore, samples of this composition require HF for quantitative microwave digestion, as it can efficiently dissolve silicates [46]. HF is a strong complexing agent that can increase the solubility and stability of certain metals. However, using HF can cause problems with laboratory equipment because it is corrosive to glass and quartz. During the study, the most common method used, which is the evaporation of acid residues after digestion, was avoided. This was particularly important given that one of the analytes was mercury. The acid was used at a relatively low concentration when taking into account the final dilution and sample preparation processes, which were conducted in a closed system. This did not negatively affect the spectrometer elements or pose a hazard in the laboratory. The use of microwave-assisted digestion allowed for a short digestion time and high efficiency of the procedure. Before further analysis, the digested solutions were diluted according to the concentrations of the elements determined. Corresponding blanks were also prepared using the above microwave digestion procedure to correct for possible contamination from reagents used in sample preparation. Pb, Cd, and Hg were determined immediately after sample preparation.

### 3.4. Methods of Analysis

#### 3.4.1. Spectrophotometric Analysis

MeCbl and CNCbl in dietary supplements were determined using a UV-Vis spectrophotometer from Analytik Jena (Jena, Germany), model Specord 50 Plus. The spectrophotometer is characterized by a wide wavelength range from 190 to 1100 nm. This device is equipped with a halogen and a deuterium lamp as the radiation sources, and the detector is in the form of two photodiodes.

MeCbl and CNCbl were determined by UV-Vis spectrophotometry in samples after ultrasound-assisted extraction. All measurements were performed in standard 10 mm quartz cells. Calibration was performed using the standard calibration technique. The samples were diluted before analysis according to the initial concentration of the analyte. The maximum of absorbance for CNCbl was set to 361 nm and for MeCbl to 351 nm, and their determination was performed using a multicomponent analysis. No significant differences were observed in the spectra obtained for the standards and analytical samples at both wavelengths, indicating that there are no substantial interferences.

#### 3.4.2. LC-MS/MS Analysis

The determination of MeCbl and CNCbl was carried out using a high-performance UltiMate 3000 RSLC chromatographic system from Dionex (Sunnyvale, CA, USA) coupled to an API 4000 QTRAP triple quadrupole mass spectrometer from AB Sciex (Foster City, CA, USA).

Chromatographic analysis was performed on samples after ultrasound-assisted extraction by injecting 5 µL of the samples into a Kinetex Evo C18 column (150 mm × 2.1 mm I.D.; 2.6 μm) from Phenomenex (Torrance, CA, USA) maintained at 35 °C. The analysis was performed in a gradient of mobile phases A (20 mM ammonium formate) and B (acetonitrile) flowing at 0.3 mL min^−1^ in the following gradient (0 min 10% B; 3 min 100% B). The effluent from the analytical column was introduced into the electrospray ionization source of the mass spectrometer operating in positive ion mode. The electrospray source parameters were as follows: curtain gas pressure, 20 psi; nebulizing gas pressure, 45 psi; auxiliary gas pressure, 45 psi; source temperature, 450 °C; ESI voltage, 4500 V; and collision gas flow was set to medium level. The dwell time for each transition in the multiple reaction monitoring mode was set to 200 ms. MeCbl was quantified using the transition from the ion at *m/z* = 665 to *m/z* = 636 (at collision energy CE = 26 eV, entrance potential EP = 4 V, and cell exit potential CXP = 17 V). Confirmatory transition was from *m/z* = 665 to *m/z* = 147 (at CE = 45 eV, EP = 3 V, and cell exit potential CXP = 7 V). CNCbl was quantified using the transition from *m/z* = 678 to *m/z* = 147 (at CE = 45 eV, EP = 4 V, and CXP = 7 V). Confirmatory transition was from *m/z* = 678 to *m/z* = 359 (at CE = 33 eV, EP = 5 V, and CXP = 8 V). Calibration was performed by the standard calibration technique. Prior to quantification, samples were diluted according to the concentration of the analytes.

#### 3.4.3. HR-CS ET AAS Analysis

An Analytik Jena ContrAA 700 high-resolution atomic absorption spectrometer (HR-CS ET AAS, Analytik Jena, Jena, Germany) was used for the determination of Cd and Pb in supplements. The spectrometer was equipped with a 300 W xenon short-arc lamp as a continuum radiation source, a compact high-resolution double echelle monochromator, and a charge-coupled device (CCD) array detector with a resolution of about 2 pm per pixel in the far-ultraviolet range. As a graphite atomizer, a pyrolytically coated graphite tube was used.

To determine the Cd and Pb contents, the dried samples (ca. 0.3 g) were digested with a microwave digestion system. In the course of analysis, 20 μL of the sample solution and 5 μL of the modifier solution were injected into a pyrolytically coated graphite tube. The standard calibration technique was used to determine Cd and Pb in vitamin B12 supplements. The study was carried out under optimum conditions as summarized in Table 4. During the AAS analysis, the tube was dried in three stages at temperatures of 80 °C, 90 °C, and 120 °C. After atomization, the tube was cleaned in the “cleanout” stage at 2250 °C.

#### 3.4.4. Total Hg Determination

The determination of Hg was carried out by the cold-vapor generation technique with atomic absorption detection (CV AAS). For this purpose, a mercury analyzer (Model Aula-254, Mercury Instruments, GmbH, Karlsfeld, Germany) was used. An Hg electrodeless low-pressure mercury discharge lamp (EDL) was used as the radiation source. Instrumental parameters were set up as follows: wavelength, 253.65 nm; spectral bandpass, 0.4 nm; quartz cell temperature, 50 °C; measurement mode, peak height; background correction, deuterium lamp. A thermoelectric gas dehumidifier and heating of the optical cell eliminated the moisture and prevented interference from water vapor.

To determine the Hg content, the dried samples (ca. 0.3 g) were digested with a microwave digestion system. The determination was performed by the reduction in all Hg species to volatile Hg(0) with SnCl_2_ and detected by AAS. During the analysis, 2.5 mL of the solution, containing the digested sample, was made up the volume to 10 mL in a calibrated flask with water. Then, 5 mL of this solution was placed in the reaction flask for vapor generation. Hg vapor was generated in 2 mol L^−1^ HCl medium using 2% (*w/v*) SnCl_2_ reducing agent. Hg(0) was transferred to the quartz cell by an Ar stream (70 mL min^−1^). The total Hg concentration of the samples was determined by the standard calibration technique using CV AAS.

### 3.5. Health Risk Assessment by Hazard Quotients

The human health risk due to chronic exposure to heavy metals was assessed using hazard quotients [14]. The hazard quotient (HQ) is a ratio used to evaluate the likelihood of health issues other than cancer arising from exposure to a substance. It is calculated by dividing the estimated exposure level (e.g., the average daily intake) by the reference dose (RfD), the level of exposure believed to be safe. An HQ of 1 or less suggests that adverse health effects are unlikely. An HQ greater than 1 indicates the potential for adverse effects, although the extent of the risk is not specified [47].

The hazard quotients were computed based on the average daily dose and the reference dose as given in Equation (1):

HQ = ADD/RfD,(1)

where ADD is the average daily dose (mg kg^−1^ day^−1^) and RfD is the reference dose (mg kg^−1^ day^−1^).

The average daily dose was calculated using Equation (2):

ADD = (C∙IR)/BW,(2)

where C is the concentration of a metal in a tablet or a capsule (µg g^−1^); IR is the ingestion rate, i.e., mass of tablets or capsule fill consumed per day (g day^−1^); and BW is the body weight of an average person (kg).

The RfD may be calculated based on the no-observed-adverse-effect level found in the literature [23], but for many contaminants, it may be found in the literature. In the present study, the RfD values used were based on the literature reports. For Cd, the RfD was 0.0005 mg kg^−1^ day^−1^ [23] and for Pb, it was 0.0014 mg kg^−1^ day^−1^ [45].

To summarize the effect of Cd and Pb, the hazard index was calculated, which is the sum of the HQ values calculated for the particular supplement [45].

## 4. Conclusions

Analytical methods for determining vitamin B12 and heavy metals in dietary supplements have been developed. The results obtained using an inexpensive spectrophotometric method were found to be consistent with those obtained using the LC-MS/MS technique. This enables inexpensive and rapid verification of the vitamin B12 content of simple dietary supplements that contain vitamin B12 as their only active ingredient.

Most of the supplements tested contained the declared amount of vitamin B12, although one of them contained a different form of the vitamin than that declared on the packaging, i.e., methylcobalamin instead of cyanocobalamin. Unfortunately, in 2 out of 10 samples, significantly lower vitamin B12 content was recorded, approximately 39% and 41%. Such low levels compared to those declared by manufacturers may be a cause for concern for consumers, as they need adequate vitamin B12 supplementation.

The heavy metal content in the samples of the supplements tested was not excessively high. Although cadmium and lead were detected in almost all supplements, their content does not pose a risk to consumers. Both the hazard quotient and hazard index values calculated for all 10supplements were below 1. This indicates that there is no significant risk to consumers associated with the consumption of the supplements tested.

Future research should focus on developing methods to verify the presence of other contaminants in dietary supplements. These methodologies should be designed to be incorporated into the regular work of pharmaceutical and food laboratories.

## Figures and Tables

**Table 1:** The results of MeCbl and CNCbl determination in vitamin B12 supplements (average value ± standard deviation for five replicate measurements). AI and RDA values are also presented for comparison.

Supplement	MeCbl Labeled (μg/Tablet (Capsule))	CNCbl Labeled (μg/Tablet (Capsule))	Determined MeCbl (μg/Tablet (Capsule))		Determined CNCbl (μg/Tablet (Capsule))		Percentage of Declared Content Based on	
			UV-Vis Detection	LC-MS/MS Detection	UV-Vis Detection	LC-MS/MS Detection	UV-Vis Results (%)	LC-MS/MS Results (%)
M1	500	-	194.9 ± 5.8	197.1 ± 15.5	-	-	39	39
M2	500	-	419.5 ± 9.9	435.0 ± 32.2	-	-	84	87
M3	100	-	96.9 ± 6.1	93.5 ± 1.8	-	-	97	94
M4	250	-	111.6 ± 4.8	102.4 ± 6.8	-	-	45	41
M5	400	-	335.6 ± 9.6	324.3 ± 29.7	-	-	84	81
M6	100	-	121.9 ± 3.3	108.1 ± 4.3	-	-	122	108
C1	-	10	7.2 ± 0.5	6.1 ± 0.1	0.9 ± 0.1	0.6 ± 0.1	81	67
C2	-	10	-	-	10.6 ± 0.7	12.7 ± 0.2	106	127
C3	-	100	-	-	110.6 ± 5.5	104.6 ± 3.5	111	105
C4	-	500	-	-	394.2 ± 15.6	429.2 ± 17.8	79	86

**Table 2:** The results of Cd, Hg, and Pb determination in vitamin B12 supplements (average value ± standard deviation for five replicate measurements).

Supplement	Cd (mg kg^−1^ d.m.)	Pb (mg kg^−1^ d.m.)	Hg (mg kg^−1^ d.m.)
M1	0.14 ± 0.01	0.17 ± 0.02	<LOD
M2	<LOD	0.16 ± 0.01	<LOD
M3	0.38 ± 0.08	0.43 ± 0.02	<LOD
M4	0.21 ± 0.02	0.14 ± 0.02	<LOD
M5	0.26 ± 0.03	0.23 ± 0.01	<LOD
M6	0.19 ± 0.03	<LOD	<LOD
C1	0.27 ± 0.02	0.19 ± 0.01	<LOD
C2	0.45 ± 0.03	0.28 ± 0.03	<LOD
C3	0.37 ± 0.02	0.17 ± 0.02	<LOD
C4	<LOD	0.22 ± 0.01	<LOD

d.m.—dry mass, LOD—the limit of detection.

**Table 3:** A risk assessment calculated due to the presence of heavy metals in the tested supplements.

Supplement	Hazard Quotient for Cd	Hazard Quotient for Pb	Hazard Index
M1	8.60 × 10^−4^	3.73 × 10^−4^	1.23 × 10^−3^
M2	- ^1^	8.44 × 10^−4^	8.44 × 10^−4^
M3	2.80 × 10^−3^	1.13 × 10^−3^	3.94 × 10^−3^
M4	9.26 × 10^−4^	2.21 × 10^−4^	1.15 × 10^−3^
M5	1.53 × 10^−3^	4.82 × 10^−4^	2.01 × 10^−3^
M6	2.45 × 10^−3^	- ^1^	2.45 × 10^−3^
C1	1.70 × 10^−3^	4.28 × 10^−4^	2.13 × 10^−3^
C2	1.57 × 10^−3^	3.49 × 10^−4^	1.92 × 10^−3^
C3	3.28 × 10^−3^	5.37 × 10^−4^	3.81 × 10^−3^
C4	- ^1^	1.11 × 10^−3^	1.11 × 10^−3^

^1^ No Cd was detected in samples M2 and C4, and no Pb in sample M6.

**Table 4:** Experimental conditions for the determination of metal impurities (Cd and Pb) in vitamin B12 supplements using HR-CS ET AAS detection.

HR-CS ET AAS Detection		
Parameter	Cd	Pb
Wavelength (nm)	228.8	283.3
Lamp current (A)	9	
Spectral range (pixel)	200	
Dispersion (pm pixel^−1^)	2	
Read time (s)	5	
Delay time (s)	0	
Measurement mode	peak height	
Sample volume (μL)	20	
Modifier/concentration (g L^−1^)	Mg(NO_3_)_2_/0.2	NH_4_H_2_PO_4_/1.0
Modifier volume (μL)	5	
Furnace program main steps		
Pyrolysis	1000 °C	1300 °C
	ramp 300 °C s^−1^, hold 10 s	
Atomization	1500 °C,	2200 °C
	ramp 2500 °C s^−1^, hold 5 s	

## Data Availability

All relevant data are included in this article and presented as figures and tables. Other data are available from the corresponding author upon reasonable request.

## References

[1] Zhou Y., He A., Xu B. (2025). Natural Resources, Quantification, Microbial Bioconversion, and Bioactivities of Vitamin B12 for Vegetarian Diet. Food Chem..

[2] Dinu M., Abbate R., Gensini G.F., Casini A., Sofi F. (2017). Vegetarian, Vegan Diets and Multiple Health Outcomes: A Systematic Review with Meta-Analysis of Observational Studies. Crit. Rev. Food Sci. Nutr..

[3] National Institutes of Health Dietary Supplement Fact Sheets, Vitamin B12. https://ods.od.nih.gov/factsheets/VitaminB12-HealthProfessional/.

[4] Doets E.L., In’t Veld P.H., Szczecińska A., Dhonukshe-Rutten R.A., Cavelaars A.E., van’t Veer P., Brzozowska A., de Groot L.C. (2013). Systematic Review on Daily Vitamin B12 Losses and Bioavailability for Deriving Recommendations on Vitamin B12 Intake with the Factorial Approach. Ann. Nutr. Metab..

[5] Stabler S.P., Allen R.H. (2004). Vitamin B12 Deficiency as a Worldwide Problem. Annu. Rev. Nutr..

[6] Bakaloudi D.R., Halloran A., Rippin H.L., Oikonomidou A.C., Dardavesis T.I., Williams J., Wickramasinghe K., Breda J., Chourdakis M. (2021). Intake and Adequacy of the Vegan Diet. A Systematic Review of the Evidence. Clin. Nutr..

[7] EFSA Panel on Dietetic Products, Nutrition, and Allergies (NDA) (2015). Scientific Opinion on Dietary Reference Values for Cobalamin (Vitamin B12). EFSA J..

[8] Fernandes S., Oliveira L., Pereira A., Costa M.d.C., Raposo A., Saraiva A., Magalhães B. (2024). Exploring Vitamin B12 Supplementation in the Vegan Population: A Scoping Review of the Evidence. Nutrients.

[9] Smith A.D., Warren M.J., Refsum H., Eskin N.A.M. (2018). Vitamin B12. Advances in Food and Nutrition Research.

[10] Allen L.H., Coates P.M., Betz J.M., Blackman M.R., Cragg G.M., Levine M., Moss J., White J.D. (2010). Vitamin B12. Encyclopedia of Dietary Supplements.

[11] van Kapel J., Spijkers L.J.M., Lindemans J., Abels J. (1983). Improved Distribution Analysis of Cobalamins and Cobalamin Analogues in Human Plasma in Which the Use of Thiol-Blocking Agents Is a Prerequisite. Clin. Chim. Acta.

[12] Augustsson A., Qvarforth A., Engström E., Paulukat C., Rodushkin I. (2021). Trace and Major Elements in Food Supplements of Different Origin: Implications for Daily Intake Levels and Health Risks. Toxicol. Rep..

[13] Seremet O., Olaru O., Gutu C., Nitulescu G., Ilie M., Negres S., Zbarcea C., Purdel C., Spandidos D., Tsatsakis A. (2018). Toxicity of Plant Extracts Containing Pyrrolizidine Alkaloids Using Alternative Invertebrate Models. Mol. Med. Rep..

[14] Amariei S., Gutt G., Oroian M. (2017). Study on Toxic Metal Levels in Food Supplements. Rev. De Chim..

[15] Santos A.J.M., Khemiri S., Simões S., Prista C., Sousa I., Raymundo A. (2024). Determination of Cobalamin (Vitamin B12) in Selected Microalgae and Cyanobacteria Products by HPLC-DAD. J. Appl. Phycol..

[16] Heudi O., Kilinç T., Fontannaz P., Marley E. (2006). Determination of Vitamin B12 in Food Products and in Premixes by Reversed-Phase High Performance Liquid Chromatography and Immunoaffinity Extraction. J. Chromatogr. A.

[17] Santos A.J.M., Khemiri S., Simões S., Prista C., Sousa I., Raymundo A. (2023). The Importance, Prevalence and Determination of Vitamins B6 and B12 in Food Matrices: A Review. Food Chem..

[18] (2022). International Conference on Harmonisation of Technical Requirements for Registration of Pharmaceuticals for Human Use.

[19] (2024). 232 Elemental Impurities-Limits.

[20] (2023). 2232 Elemental Contaminants in Dietary Supplements.

[21] Sheth A.C., Patel P.U. (2020). Review of Elemental Impurities in Pharmaceuticals Arena. Int. J. Pharm. Qual. Assur..

[22] Abernethy D.R., DeStefano A.J., Cecil T.L., Zaidi K., Williams R.L. (2010). Metal Impurities in Food and Drugs. Pharm. Res..

[23] Uslu İ., Alp O., Karahalil B. (2025). Monitoring of Essential and Toxic Elements in Multivitamin/Mineral Effervescent Tablet Supplements and Safety Assessment. Biol. Trace Elem. Res..

[24] Ćwieląg-Drabek M., Piekut A., Szymala I., Oleksiuk K., Razzaghi M., Osmala W., Jabłońska K., Dziubanek G. (2020). Health Risks from Consumption of Medicinal Plant Dietary Supplements. Food Sci. Nutr..

[25] Poniedziałek B., Niedzielski P., Kozak L., Rzymski P., Wachelka M., Rzymska I., Karczewski J., Rzymski P. (2018). Monitoring of Essential and Toxic Elements in Multi-Ingredient Food Supplements Produced in European Union. J. Consum. Prot. Food Saf..

[26] Korfali S.I., Hawi T., Mroueh M. (2013). Evaluation of Heavy Metals Content in Dietary Supplements in Lebanon. Chem. Cent. J..

[27] Bernhardt C., Zhu X., Schütz D., Fischer M., Bisping B. (2019). Cobalamin Is Produced by Acetobacter Pasteurianus DSM 3509. Appl. Microbiol. Biotechnol..

[28] (2019). Appendix K: Guidelines for Dietary Supplements and Botanicals.

[29] Nutrition Labelling Compliance Test. https://inspection.canada.ca/en/food-labels/labelling/industry/nutrition-labelling/additional-information/compliance-test#c4.

[30] Crawford C., Lindsey A.T., Avula B., Katragunta K., Khan I.A., Deuster P.A. (2024). Label Accuracy and Quality of Select Weight-Loss Dietary Supplements Sold on or near US Military Bases. Nutrients.

[31] Werner J., Frankowski R., Grześkowiak T., Zgoła-Grześkowiak A. (2021). High-Performance Liquid Chromatography with Fluorescence Detection for the Determination of Capsaicin and Dihydrocapsaicin in Fat-Burning Dietary Supplements. Anal. Lett..

[32] You H., Gershon H., Goren F., Xue F., Kantowski T., Monheit L. (2022). Analytical Strategies to Determine the Labelling Accuracy and Economically-Motivated Adulteration of “Natural” Dietary Supplements in the Marketplace: Turmeric Case Study. Food Chem..

[33] Starr R.R. (2015). Too Little, Too Late: Ineffective Regulation of Dietary Supplements in the United States. Am. J. Public Health.

[34] Walczak P. (2021). Informacja o Wynikach Kontroli Wprowadzenie Do Obrotu Suplementów Diety.

[35] Paul C., Brady D.M. (2017). Comparative Bioavailability and Utilization of Particular Forms of B12 Supplements With Potential to Mitigate B12-Related Genetic Polymorphisms. Integr. Med..

[36] Obeid R., Fedosov S.N., Nexo E. (2015). Cobalamin Coenzyme Forms Are Not Likely to Be Superior to Cyano- and Hydroxyl-cobalamin in Prevention or Treatment of Cobalamin Deficiency. Mol. Nutr. Food. Res..

[37] Adams J.F., Ross S.K., Mervyn L., Boddy K., King P. (1971). Absorption of Cyanocobalamin, Coenzyme B _12_, Methylcobalamin, and Hydroxocobalamin at Different Dose Levels. Scand. J. Gastroenterol..

[38] Temova Rakuša Ž., Roškar R., Hickey N., Geremia S. (2022). Vitamin B12 in Foods, Food Supplements, and Medicines—A Review of Its Role and Properties with a Focus on Its Stability. Molecules.

[39] Freeman A.G. (1996). Hydroxocobalamin versus Cyanocobalamin. J. R. Soc. Med..

[40] Carmel R. (2008). Efficacy and Safety of Fortification and Supplementation with Vitamin B12: Biochemical and Physiological Effects. Food Nutr. Bull..

[41] Carmel R. (2008). How I Treat Cobalamin (Vitamin B12) Deficiency. Blood.

[42] Kebbekus B.B., Mitra S. (2003). Preparation of Samples for Metals Analysis. Sample Preparation Techniques in Analytical Chemistry.

[43] Krawczyk-Coda M., Pietrowski M., Stanisz E. (2025). Enhanced Determination of Iodine by Molecular Absorption Spectrometry after Dispersive Solid-Phase Extraction with Direct Quantification of Selenium in Foods. Food Chem..

[44] The European Commission (2023). Commission Regulation (EU) 2023/915 of 25 April 2023 on Maximum Levels for Certain Contaminants in Food and Repealing. EUR-Lex Regulation (EC) No 1881/2006. Off. J. Eur. Union.

[45] Nguyen K.T., Nguyen H.M., Truong C.K., Ahmed M.B., Huang Y., Zhou J.L. (2019). Chemical and Microbiological Risk Assessment of Urban River Water Quality in Vietnam. Environ. Geochem. Health.

[46] Pinna M., Signorelli A., Binda G., Dossi C., Rampazzi L., Spanu D., Recchia S. (2022). How to Clean and Safely Remove HF from Acid Digestion Solutions for Ultra-Trace Analysis: A Microwave-Assisted Vessel-Inside-Vessel Protocol. Methods Protoc..

[47] Wang C.-C., Zhang Q.-C., Kang S.-G., Li M.-Y., Zhang M.-Y., Xu W.-M., Xiang P., Ma L.Q. (2023). Heavy Metal(Loid)s in Agricultural Soil from Main Grain Production Regions of China: Bioaccessibility and Health Risks to Humans. Sci. Total Environ..

